# Evaluation of qualitative and semi-quantitative cut offs for rapid diagnostic lateral flow test in relation to serology for the detection of SARS-CoV-2 antibodies: findings of a prospective study

**DOI:** 10.1186/s12879-022-07786-5

**Published:** 2022-10-31

**Authors:** Maddalena Peghin, Giulia Bontempo, Maria De Martino, Alvisa Palese, Valentina Gerussi, Elena Graziano, Martina Fabris, Federica D’Aurizio, Francesco Sbrana, Andrea Ripoli, Francesco Curcio, Miriam Isola, Carlo Tascini

**Affiliations:** 1 Infectious Diseases Division, Department of Medicine, University of Udine and Azienda sanitaria universitaria Friuli Centrale (ASUFC), Udine, Italy; 2 Infectious and Tropical Diseases Unit, Department of Medicine and Surgery, University of Insubria-ASST-Sette Laghi, Varese, Italy; 3 Division of Medical Statistics, Department of Medicine (DAME), University of Udine, 33100 Udine, Italy; 4 Department of Medical Sciences, School of Nursing, University of Udine, Udine, Italy; 5 Institute of Clinical Pathology, Azienda Sanitaria Universitaria Friuli Centrale, Udine, Italy; 6 U.O. Lipoapheresis and Center for Inherited Dyslipidemias - Fondazione Toscana Gabriele Monasterio, Pisa, Italy; 7 Bioengineering Department, Fondazione Toscana Gabriele Monasterio, Pisa, Italy; 8 Infectious Diseases Division , Azienda sanitaria universitaria Friuli Centrale (ASUFC), Presidio Ospedaliero Universitario Santa Maria della Misericordia, Piazzale Santa Maria della Misericordia 15, 33010 Udine, Italy

**Keywords:** COVID-19, SARS-CoV-2, Rapid diagnostic test, Lateral flow immunoassay, Serology, Antibodies, POC, RDT, CLIA, ELISA

## Abstract

**Background:**

There is limited information to compare the qualitative and semi-quantitative performance of rapid diagnostic tests (RDT) and serology for the assessment of antibodies against severe acute respiratory syndrome coronavirus 2 (SARS-CoV-2). Therefore, the objective of the study was (a) to compare the efficacy of SARS-CoV-2 antibody detection between RDT and laboratory serology, trying to identify appropriate semi-quantitative cut-offs for RDT in relation with quantitative serology values and to (b) evaluate diagnostic accuracy of RDT compared to the NAAT gold standard in an unselected adult population.

**Methods:**

SARS-CoV-2 antibodies were simultaneously measured with lateral flow immunochromatographic assays (LFA), the Cellex qSARS-CoV-2 IgG/IgM Rapid Test (by capillary blood), the iFlash-SARS-CoV-2 IgG/IgM chemiluminescent immunoassay (CLIA) (by venous blood) and the nucleic acid amplification test (NAAT) in samples from in- and out-patients with confirmed, suspected and negative diagnosis of coronavirus disease 2019 (COVID-19) attending Udine Hospital (Italy) (March-May 2020). Interpretation of RDT was qualitative (positive/negative) and semi-quantitative based on a chromatographic intensity scale (negative, weak positive, positive).

**Results:**

Overall, 720 paired antibody measures were performed on 858 patients. The qualitative and semiquantitative agreement analysis performed in the whole sample between LFA and CLIA provided a Kendall’s tau of 0.578 (p < 0.001) and of 0.623 (p < 0.001), respectively, for IgM and IgG. In patients with a diagnosis of COVID-19, accordance between LFA and CLIA was maintained as a function of time from the onset of COVID-19 disease and the severity of disease both for qualitative and semi-quantitative assessments. RDT compared to the NAAT gold standard in 858 patients showed 78.5% sensitivity (95% CI 75.1%-81.7%) and 94.1% specificity (95% CI 90.4%-96.8%), with variable accordance depending on the timing from symptom onset.

**Conclusion:**

The RDT used in our study can be a non-invasive and reliable alternative to serological tests and facilitate both qualitative and a semi-quantitative antibody detection in COVID-19.

## Background

Access to accurate and timely diagnostic test for severe acute respiratory syndrome coronavirus 2 (SARS-CoV-2) infection plays a major role to optimise clinical care and public health management worldwide. Since the beginning of the pandemic, market was flooded with several coronavirus disease 2019 (COVID-19) diagnostic tests of different classes with variable testing validation and regulatory oversight. The performance of nucleic acid amplification test (NAAT) in respiratory samples is currently the gold standard for confirming diagnosis, while rapid SARS-CoV-2 antigen diagnostic tests are used as an alternative to NAAT, with typically lower sensitivity than NAAT [1]. In addition, SARS-CoV-2 specific antibody detection is considered to complement NAAT, particularly in the late stages of disease (3–4 weeks post-symptom onset) to identify prior or late infection for clinical and epidemiological purposes [2]. The most common current diagnostic platforms utilised for SARS-CoV-2 specific antibody detection comprise rapid diagnostic tests (RDTs) with a qualitative (or semi-quantitative) assessment of antibodies mainly based on lateral flow assays (LFA) and centralised quantitative serological laboratory testing. Unlike serological tests, RDT for anti-SARS-CoV2 antibodies have the advantage of being simple to run and interpret and can be used at point of care (POC) as an alternative to diagnostic serology facilities [3]. However, the usefulness and accuracy of RDT for antibody detection has been widely questioned, due to their general lack of quantitative data, limited sensitivity and specificity when compared to non-RDT methods. In addition, the evidence for its accuracy in COVID-19 diagnosis relies on manufacturers’ self-validation and on the literature available, which offer estimations in selected biased populations resulting from laboratory testing data sets, or subgroups of patients. A better understanding and a rigorous validation for qualitative and identification of semi-quantitative values of RDT for SARS-CoV-2 antibody may support appropriate pathways for public health planning to control the dynamic of the COVID-19 pandemic in different real-life epidemiological settings [3].

## Methods

### Aim, study setting and population

The aim of this extensive prospective study was to compare the efficacy of SARS-CoV-2 antibody detection between RDT and laboratory serology, trying to identify appropriate semi-quantitative cut-offs for RDT in relation with quantitative serology values and to evaluate diagnostic accuracy of RDT compared to the NAAT gold standard in an unselected adult population.

We completed this study at Udine Hospital (Italy), a 1,000-bed tertiary-care teaching hospital identified as a regional referral centre for COVID-19 patients and serving approximately 350,000 citizens. RDT and serology samples were simultaneously collected by trained nurses from a cohort of all consecutive adult in- and out-patients (≥ 18 years) attending the Infectious Disease Department with the diagnosis of confirmed COVID-19, suspicious COVID-19, and negative for COVID-19 (March-May 2020).

The concordance between serological and rapid tests measurements were analysed considering paired measures (same patient at the same time). Samples were collected at various phases of the follow-up after onset of symptoms as explained by the patient and divided as follow: early stage (< 15 days); late stage: between day 15 and day 30; between month 1 and 2, after month 2. A SARS-CoV-2 serological test and RDT follow-up test were performed for a subset of enrolled patients with a diagnosis of COVID 19 that accepted monthly serological controls (+/- 15 days), according to a previously established protocol. The details of this prospective cohort have been provided previously [2].

### Acute COVID-19 and baseline definitions

Diagnosis of COVID-19 infection was established as confirmed (positive SARS-CoV-2 NAAT in nasopharyngeal swabs or bronchoalveolar lavage) or suspected (negative SARS-CoV-2 NAAT in respiratory tract samples but suggestive laboratory or imaging findings and/or positive SARS-CoV-2 serology) during the acute phase of the disease. Remaining patients were classified as negative for COVID-19 [4].

Patients were classified using the COVID-19 Disease Severity Scale and specifically, for the analysis, patients were classified into three groups: (1) asymptomatic, (2) mild, and (3) moderate to critical disease [5].

### Laboratory methods

**NAAT test.** Respiratory samples were tested for SARS-CoV-2 using RT-PCR targeted that investigated the E gene for screening and then the RdRp and N genes of SARS-CoV- 2 for confirmation (Roche Respiratory Panel Assay). The specimens were considered positive if the cycle threshold (CT) value for at least one of the three genes was ≤ 36 [4].

**Lateral flow immunoassays.** Samples for LFA were obtained from one capillary blood drop (10–20 µL) obtained from a finger stick sample. The Cellex SARS-CoV-2 IgG/IgM Rapid Test is a lateral flow qualitative chromatographic immunoassay to detect IgG/IgM s against SARS-CoV-2 N and S protein with a positive percent agreement and negative percent agreement of 93.75% and 96.40% respectively [6].

**Chemiluminescent immunoassay (CLIA**). Samples for SARS-CoV-2 serologies were obtained from venous blood samples. iFlash-SARS-CoV-2 (Shenzhen Yhlo Biotech Co. Ltd. China, distributed in Italy by Pantec SRL), is a paramagnetic particle CLIA for detection of IgG/IgM against SARS-CoV-2 N and (non –RBD) S protein (cut-off for IgG/ IgM positivity > 10.0 kAU/L. The test performance has been documented to have a sensitivity and specificity of 86.1% and 99.2% for IgM and 93.7% and 96.3% for IgG, respectively [7].

### Identification of semi-quantitative cut-offs for RDT

**Fig. 1:**
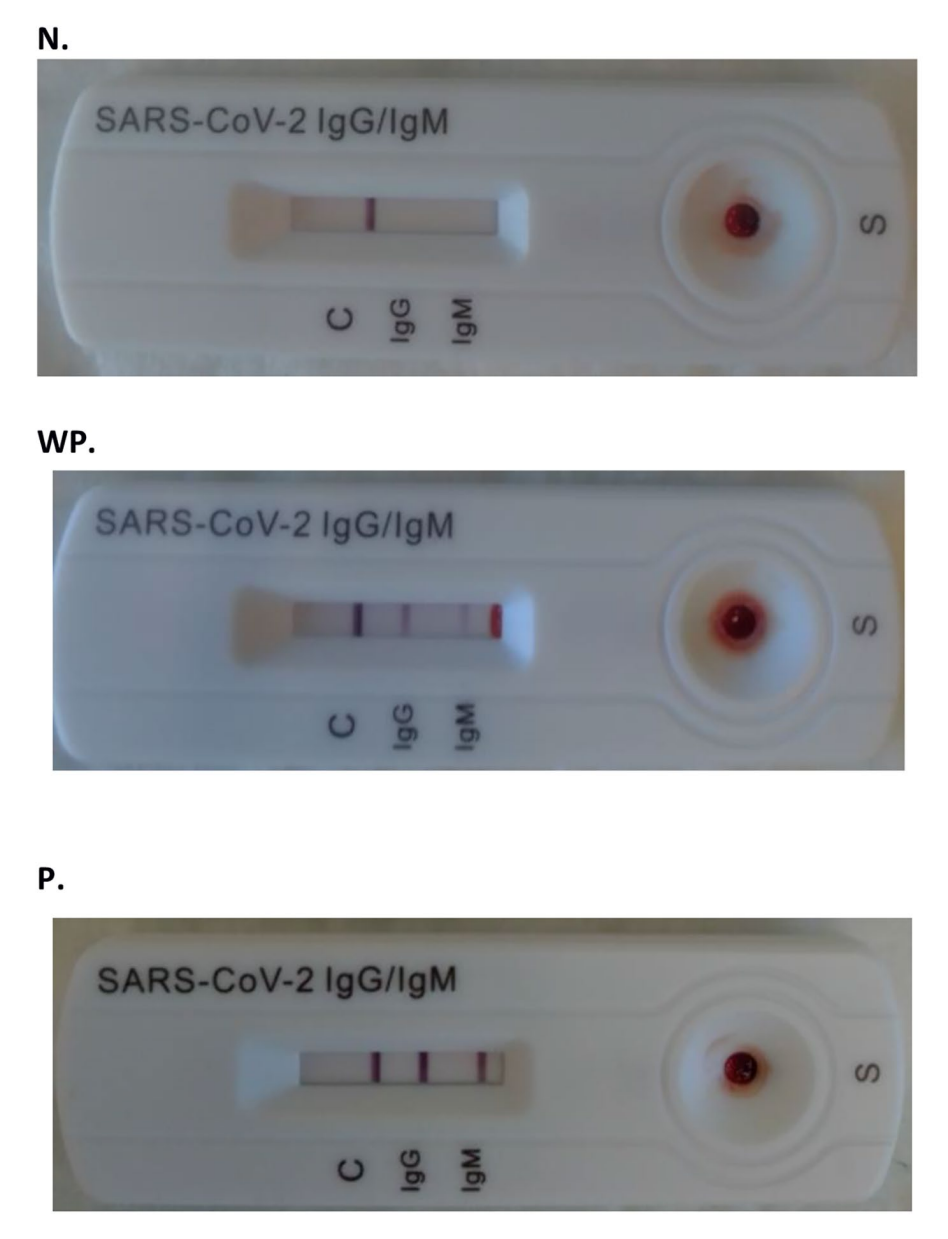
Semi quantitative chromatographic scale for RDT. Legend: C, control; N, Negative; WP, Weak positive; P, Positive

An internal scientific committee consisting of three investigators (two infectious disease specialists and one laboratory medicine specialist) the hospital developed a chromatographic intensity scale (Fig. 1) and participated in the interpretation of qualitative (positive or negative) and semi-quantitative LFA results. A picture of every rapid test was taken at the manufacturer’s established time of reading (15 min), a photographic archive of LFAs was made for every test and test results were independently reviewed by three of these investigators. Results were based on full consensus among the experts. According to the expert panel chromatographic scale, the IgG and IgM band was classified as either N, Negative; WP, Weak positive; or P, Positive. Figure 1 shows three RDT displaying an example of each possible result observed in our study.

### Primary and secondary outcomes and data collection

Primary endpoints of the study were assessed to compare the efficacy of SARS-CoV-2 antibody detection between RDT and CLIA serology in different phases and severity setting of COVID disease trying to identify appropriate semi-quantitative cut-offs for RDT in relation with quantitative serology values. The secondary endpoint was to evaluate the diagnostic accuracy of RDT compared to the NAAT gold standard.

### Statistical analysis

Descriptive statistics for categorical variables are presented as number (percent) and for continuous variables as mean ± standard deviation (SD) or median (interquartile range (IQR)). Normality was assessed using the Shapiro-Wilk test.

The relation between serological measurements and semi-quantitative interpretation of RDT was estimated using the Kruskal-Wallis test, while the relation between serological measurements and qualitative interpretation of RDT was estimated using the Mann-Whitney U test. The agreement between serological and semi-quantitative interpretation of RDT was studied with Kendall’s tau. The performance of IgG serological measurements to discriminate between the two level of qualitative interpretation of RDT was determined using De Long’s non-parametric receiver operating characteristic (ROC) analysis. Sensitivity, specificity, positive and negative predictive values and their 95% confidence intervals (CI) were calculated to assess diagnostic performance of qualitative interpretation of RDT compared to NAAT. All analyses were performed by STATA 17 statistical software, and statistical significance was set at p < 0.05.

## Results

### Study population and sample collection

Overall, during the study period, 1292 patients were evaluated in our hospital and 858 were eligible: 639 (74.5%) with a diagnosis of COVID-19 infection (619 (72.1%) confirmed and 20 (2.3%) suspected) and 219 (25.5%) negative for COVID-19. The clinical and microbiological characteristics of patients with a diagnosis of COVID-19 are reported in Table 1.

The concordance between serological and rapid tests measurements were analysed considering 720 paired measures. The average time from the first day of reported symptoms to the performance of combined RDT and serological test has a median of 44 days (range 0-133).

Samples were collected at various stages of the follow-up after onset of symptoms as declared by the patient: 114/720 (15.8%) early stage (< 15 days) and 71/720 (9.9%) at late stage (15–30 days), 311/720 (43.2%) between month 1 and 2, 224/720 (31.1%) between month 2 and 3.

**Table 1:** Patient’s baseline characteristics, clinical presentation in the subgroup of patients with diagnosis of COVID-19 at acute onset

	N = 639
Gender, n (%) Female Male	332 (52.0) 307 (48.0)
Age, median (IQR)	52 (38–63)
Acute COVID-19 severity^*^, n/N (%) Asymptomatic Mild Moderate Severe Critical	75/631 (11.9) 415/631 (65.8) 97/631 (15.4) 27/631 (4.3) 17/631 (2.7)
Management, n (%) Outpatients Inpatients Ward^ ICU	470 (73.6) 144 (22.5) 25 (3.9)

^ Infectious Disease or Pneumology Department

### Diagnostic accuracy of qualitative interpretation of RDT compared to serology

**Fig. 2:**
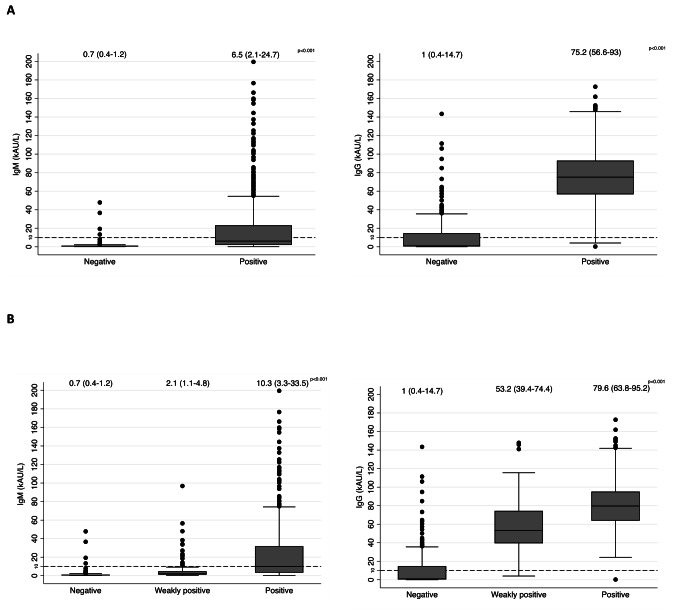
Boxplot of IgM and IgG serological measurements across the groups identified by the results of rapid tests in relation with a (A) qualitative (positive-negative) or (B) semiquantitative interpretation (N – Negative test; WP – Weak positive test; P – Positive test). Legend: Bold line reports the 10 U/ml definite as cutoff for negative serological tests. Comparisons were made using Mann Whitney U test between two groups and Kruskal-Wallis test among three groups. Legend Fig. 2B: all pairwise comparisons have a statistical significance p < 0.001. Bonferroni correction was applied

**Fig. 3:**
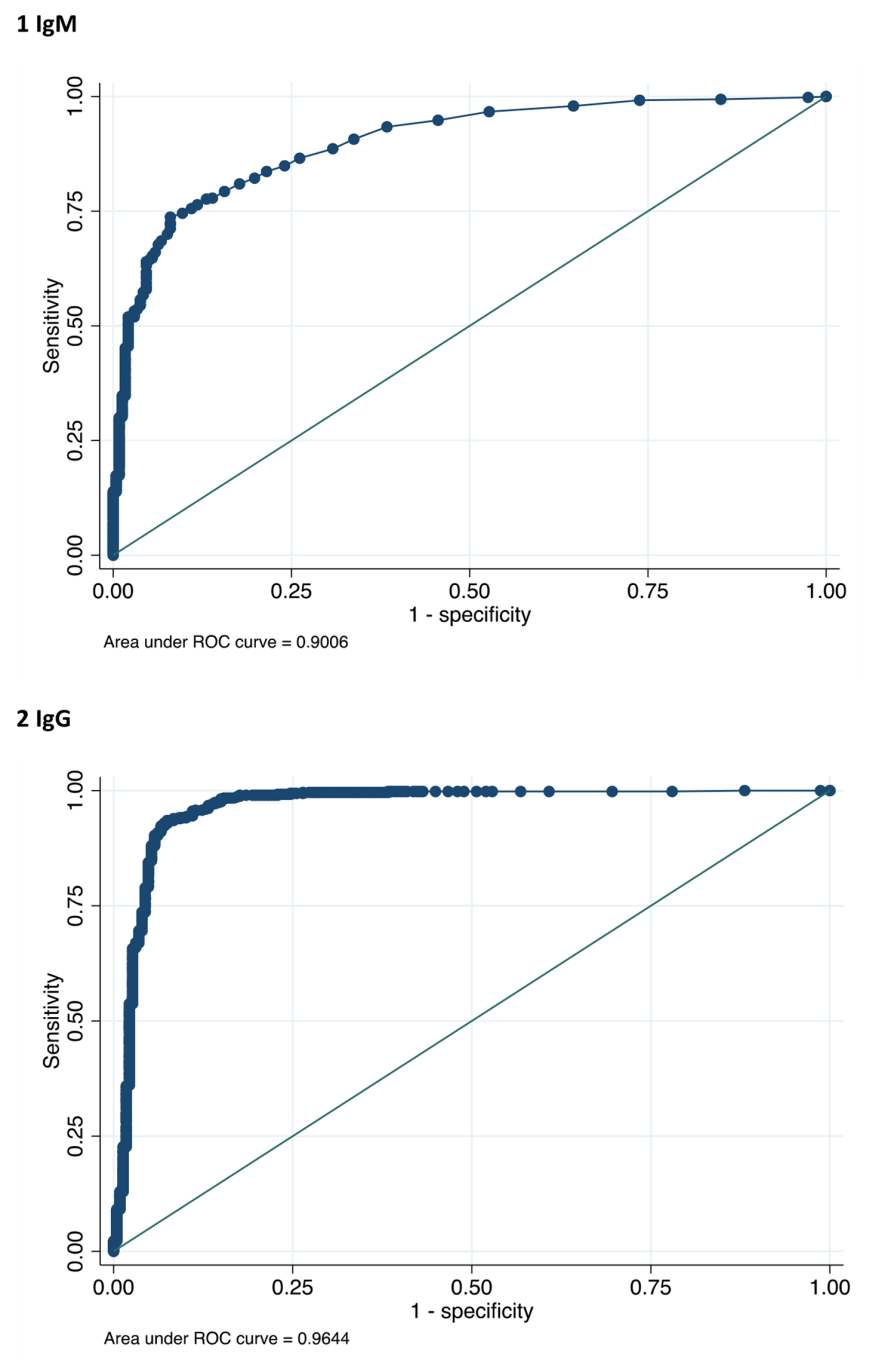
ROC curve for association of qualitative RDT compared to serology for IgM (1) and IgG (2)

Overall, when the CLIA was used as the comparator, the median between the two groups (negative and positive) was significantly different for IgM (0.7 vs. 6.5, p < 0.001) and IgG (1 vs. 75.2, p < 0.001) (Table 2). Boxplots of IgM and IgG serological measurements across the groups identified by the results of rapid tests in relation with a qualitative interpretation are described in Fig. 2A. Among patients with a diagnosis of COVID-19, qualitative RDT and serological measurements were evaluated as a function of time from the onset of COVID-19 disease and severity of disease and confirmed a significant association as described in Table 2. On the basis of the ROC curve for the association of qualitative RDT compared to serology, the AUC was 0.901 for IgM (95% CI 0.878–0.923) and 0.964 (95% CI 0.946–0.982) for IgG (Fig. 3).

**Table 2:** Serological measurements of patients with diagnosis of COVID-19, among qualitative (negative-positive) and semiquantitative (N – Negative; WP – Weak positive; P – Positive) rapid diagnostic test in function of time from the onset of COVID-19 disease and severity of disease, described ad median (IQR)

	Qualitative		p	Semiquantitative			p
	Negative	Positive		Negative	Weak positive	Positive	
**Overall**							
**IgM, median (IQR)**	0.7 (0.4–1.2)	6.5 (2.1–24.7)	**< 0.001**	0.7 (0.4–1.2)	2.1 (1.1–4.8)	10.3 (3.3–33.5)	**< 0.001**
**IgG, median (IQR)**	1 (0.4–14.7)	75.2 (56.6–93)	**< 0.001**	1 (0.4–14.7)	53.2 (39.4–74.4)	79.6 (63.8–95.2)	**< 0.001**
**Time from the onset of COVID-19**							
**IgM, median (IQR)**							
**0–15 days**	0.7 (0.4–1.1)	4.5 (1.9–9.8)	**< 0.001**	0.7 (0.4–1.1)	4.1 (1.2–5.1)	5.0 (2.1–36.5)	**< 0.001**
**15–30 days**	0.6 (0.3–1.4)	10 (4–34)	**< 0.001**	0.6 (0.3–1.4)	3.5 (2.8–7.8)	14.2 (6.2–44)	**< 0.001**
**30–60 days**	0.6 (0.4–1.1)	7.1 (2.1–28.4)	**< 0.001**	0.6 (0.4–1.1)	1.7 (1.1-4)	10.7 (2.8–37.7)	**< 0.001**
**> 60 days**	0.7 (0.5–1.3)	5.1 (1.6–19.8)	**< 0.001**	0.7 (0.5–1.3)	1.7 (0.9–4.8)	8.3 (3.3–24.7)	**< 0.001**
**IgG, median (IQR)**							
**0–15 days**	0.5 (0.3-1)	75.3 (51-96.4)	**< 0.001**	0.5 (0.3-1)	51.1 (38.8–85.3)	81.3 (56.7–97.4)	**< 0.001**
**15–30 days**	1.45 (0.45–27.7)	75.3 (55-105.3)	**< 0.001**	1.45 (0.45–27.7)	74.4 (45-105.3)	75.9 (57.1–107)	**< 0.001**
**30–60 days**	5.4 (0.5–22.8)	75.1 (58.6–92.7)	**< 0.001**	5.4 (0.5–22.8)	57.7 (45-70.4)	78.7 (65.5–94.7)	**< 0.001**
**> 60 days**	10.8 (1.3–20.6)	75.3 (53.6–91.5)	**< 0.001**	10.8 (1.3–20.6)	47.0 (28-58.3)	81.5 (64.5–94.4)	**< 0.001**
**Severity of disease**							
**IgM, median (IQR)**							
**Asymptomatic**	0.6 (0.4–1.1)	2.8 (1.0-9.7)	**< 0.001**	0.6 (0.4–1.1)	1.3 (0.7–2.3)	4.2 (1.7–25.8)	**< 0.001**
**Mild**	0.7 (0.5–1.3)	4.4 (1.7–13.9)	**< 0.001**	0.7 (0.5–1.3)	2.1 (1.1–4.6)	7.5 (2.7–22.6)	**< 0.001**
**Moderate-severe**	0.6 (0.4–0.7)	21.9 (5.6–66.1)	**< 0.001**	0.6 (0.4–0.7)	3.8 (1.2–21.5)	23.4 (8.2–68.6)	**< 0.001**
**IgG, median (IQR)**							
**Asymptomatic**	0.6 (0.3–5.4)	77.7 (56.5–92.1)	**< 0.001**	0.6 (0.3–5.4)	56.4 (36.8–74.8)	82.1 (64.1–94.2)	**< 0.001**
**Mild**	2.4 (0.5–20.6)	74.9 (55.9–91.3)	**< 0.001**	2.4 (0.5–20.6)	51.7 (38.3–72.3)	79.4 (65.4–93.2)	**< 0.001**
**Moderate-severe**	1.3 (0.4–1.3)	77.3 (58.6–99.9)	**< 0.001**	1.3 (0.4–1.3)	60.7 (48.6–83.1)	78.4 (59-100.6)	**< 0.001**

### Diagnostic accuracy of semi-quantitative interpretation of RDT compared to serology

Overall, the distribution of IgM and IgG and across the three levels of semi-quantitative interpretation of RDT in relation with serological response had a highly significant trend, as reported in Fig. 2B. The agreement analysis performed in the whole sample furnished a Kendall’s tau of 0.578 (p < 0.001) and 0.623 (p < 0.001), respectively, for IgM and IgG.

Among patients with a diagnosis of COVID-19, differences between the RDT measurement classes and CLIA test were statistically significant in relation to the time from symptoms onset (Table 2). Regarding the measurement obtained during follow-up, the Kendall’s tau was 0.392 (p < 0.001) for IgM and 0.480 (p < 0.001) for IgG between day 0–15 from the onset of COVID-19 disease, 0.671 (p < 0.001) for IgM and 0.574 (p < 0.001) for IgG between day 15–30, 0.537 (p < 0.001) for IgM and 0.524 (p < 0.001) for IgG between day 30–60, and 0.534 (p < 0.001) for IgM and 0.617 (p < 0.001) for IgG after 2 months. Furthermore, considering the severity of disease, the Kendall’s tau was 0.485 (p < 0.001) for IgM and 0.623 (p < 0.001) for IgG for asymptomatic patients, 0.537 (p < 0.001) for IgM and 0.606 (p < 0.001) for IgG for patients with mild disease and 0.355 (p < 0.001) for IgM and 0.285 (p < 0.001) for IgG for patients with moderate to severe disease (Table 2).

### Diagnostic accuracy of RDT compared to NAAT

Overall, IgG RDT qualitative accuracy compared to the NAAT gold standard showed that RDT provided an overall agreement of 82.9%, sensitivity of 78.5% (95% CI 75.1%-81.7%), specificity of 94.1% (95% CI 90.4%-96.8%), a positive predictive value (PPV) of 97.2% (95% CI 95.3%-98.5%) and a negative predictive value (NPV) of 62.8% (95% CI 57.6%-67.9%). The accuracy of RDT was related to the delay between a positive NAAT and RDT showing a sensitivity of 37.2% (95% CI 23%-53.3%) and specificity of 97.8% (95% CI 94.6%-99.4%) between day 0–15 from the onset of COVID-19 disease, a sensitivity of 71.2% (95% CI 58.7%-81.7%) and specificity of 100% (95% CI 63.1%-100%) between day 15–30, a sensitivity of 84% (95% CI 79.4%-88%) and specificity of 76.9% (95% CI 56.4%-91%) between day 30–60, and a sensitivity of 81.4% (95% CI 75.5%-86.4%) and specificity of 78.9% (95% CI 54.4%-93.9%) after two months.

## Discussion

In this prospective real life cohort study, we compared the SARS-COV-2 diagnostic test accuracy of LFA and CLIA serologic tests and demonstrated that RDT can be a non-invasive and reliable alternative to laboratory tests and facilitate not only qualitative but also semi-quantitative antibody detection. In addition, RDT accuracy compared to the NAAT gold standard showed low sensitivity and high specificity, with variable rates depending on the delay between the positive NAAT and the RDT. The strengths of the present study reside in the large cohort analysed, paired systematic collection of capillary blood obtained by finger stick for RDT and venous samples for serology, clinical characterisation of study subjects with different grades of clinical severity and monitoring over time after disease onset compared to previous literature in this line [8].

The easy-to-use SARS-COV-2 IgG/IgM combined RDT allow a valid, reproducible, cheap and non-invasive immunoassay device that can also be used in low income countries [9]. However, the usefulness and accuracy of RDT for Ab detection has been widely questioned. Our data suggests that the RDT used in our study has a high estimated qualitative agreement compared with CLIA serology, which are significantly higher compared to variable data reported by the manufacturer and to previous literature in this line, testing various RDT [1]. Furthermore, in our cohort, this agreement was maintained as a function of time from acute disease onset and the severity of COVID-19. Contradictory results of previous studies on RDT performance may be explained by the heterogeneity of different factors, including the severity of the disease, the timing of detection after the disease onset and sample types. In addition, in our study, we used RDT and CLIA assays the detecting antibody response against a combination of the S and N proteins, which is expected to improve diagnostic accuracy [10, 11].

The quantity of Ab may condition the final immunological response [2]. Of interest that, although RDT are considered only qualitative (positive/negative) tests, on the basis of previous studies [12], we created a three-level chromatographic intensity scale and found that the overall semi-quantitative concordance rate of antibodies between the LFA and CLIA was high both for IgM and for IgG and maintained as a function of time from the onset and severity of acute COVID-19. Our study suggests that RDT semi-quantitative results could be an interesting and valuable tool to offer a simple POC strategy for large-scale assessments of previous SARS-CoV-2 infection and vaccination planning (for LFA with receptor binding domain S protein target), allowing for easy monitoring of the serological response, especially in areas with limited resources [13].

Accordingly, even if serological tests for SARS-CoV-2 should not be used to diagnose acute infection a combination of semiquantitative RDT and laboratory serological testing may be an alternative two-step algorithm, as recommended by international guidelines for maximising sensitivity and specificity of SARS-CoV-2 serological diagnostics [14]. Moreover pre-test probability should be considered in the execution and interpretation of the RDT [3]. A future prospective study could use artificial intelligence or mobile phone algorithm-based interpretation of the semi-quantitative results to decrease interobserver variability and perform population-wide testing [15].

RDT accuracy compared to the NAAT gold standard showed low sensitivity and high specificity. On the basis of international regulatory agencies, RDT must have at least 80% sensitivity and 98% specificity, as compared with a reference standard of laboratory-based RT-PCR testing to be approved [16]. Previous studies addressing the performance of RDTs compared to the reference NAAT have been published with variable results, depending on severity of disease and timing from acute onset [12, 17]. The timing of antibody detection after disease onset has a crucial role, since the sensitivity of serological tests depends heavily on the delayed humoral responses. In our cohort, most COVID-19 cases were submitted to Ab detection at a late (> 15 days) stage of disease after the onset of symptoms [2]. In addition we evaluated a wide spectrum of individuals ranging from asymptomatic to severely infected who recovered from COVID-19 after the first wave [2]. Although NAAT is the gold standard, RDT allowed us to detect cases of suspected COVID-19 infection that were NAAT negative but antibody positive by RDT [1, 3].

Our study has several limitations. First, the performance and interpretation of RDT may be operator-sensitive. However, the health care professionals involved attended a training session prior to the beginning of the study and the test was read by three investigators who had to reach an agreement. Second, a relatively small sample of antibody was collected and reported in the early stages of infection, but most individuals were shown to have measurable antibody levels at late stages of infection [2]. Third, negative control samples were collected from patients with low clinical suspicion of COVID-19 and negative NAAT during the pandemic period, but not from serum samples retrieved from pre-pandemic period. Four, the RDT was performed on a finger stick blood sample; this this assay has been approved for venous blood samples (a drop of plasma, serum or whole blood). However, previous studies showed that capillary blood shows similar sensitivity for detecting anti-SARS-CoV-2 IgM and IgG antibodies as venous blood samples, allowing the use of a non-invasive immunoassay device in daily practice [18]. Finally, our results only apply to one RDT and one CLIA test, which may have an assay-dependent rate of sensitivity, specificity and antibody decline. In addition, the emergence of SARS-CoV-2 variants of concern and vaccine-induced immunity may condition different humoral responses.

## Conclusion

The selected RDT may be a useful qualitative and semi-quantitative tool for diagnosis assessing SARS-CoV-2 serology and provides valuable information on past exposure and monitoring for patients with different grades of clinical severity and variable follow-up after disease onset. Further studies are necessary to validate RDT to understand its value in establishing determining the general population’s immunological status and in assessing immunity after vaccination.

## Data Availability

All datasets used and analysed during the current study are available from the corresponding author on reasonable request.

## List of abbreviations

C: control
CLIA: chemiluminescent immunoassays
COVID-19: coronavirus disease 2019
ELISA: enzyme linked immunosorbent assays
IQR: Interquartile range
HP: High positive
LFA: lateral flow assays
N: negative
NAAT: nucleic acid amplification tests
NPV: negative predictive value
P: positive
POC: point of care
PPV: positive predictive value
RDT: rapid diagnostic test
SARS-CoV-2: severe acute respiratory syndrome coronavirus 2
SD: standard deviation
WP: weak positive
